# Topics of the Month

**Published:** 1896-03

**Authors:** 


### TOPICS OF THE MONTH.

The health commissioner of Brooklyn deserves the commendation of all good citizens in his bailiwick. He has, according to the *Medical Record*, sent a communication to the pastors of churches having bells, requesting them and all church authorities exercising control over the matter to prevent the ringing of bells before 7 o'clock A. M., and in localities where complaints of invalids are brought to their notice, to restrict bell-ringing in the day-time to as few strokes as possible.

It appears there is an ordinance in Brooklyn which expressly prohibits the ringing of bells in such a manner as to become a prejudice or peril to the life or health of individuals. Speed the day when bell-ringing and other unnecessary noises may be restricted if not prevented in Buffalo!

---

The office of coroner in this state has, in many instances, become a reproach to good government. The civilisation of the present has outlived the original purposes of the office, and while there are many conscientious coroners who do all they can to aid in the detection of crime, there are still others, especially in large cities, who fail in this respect.

Dr. W. G. Macdonald, of Albany, in a communication to the *New York Herald*, published February 14, 1896, cited a number of instances within his personal knowledge where justice has miscarried by reason of inefficient or neglectful administration of the office.

A bill was introduced in the assembly by Mr. Robbins, of Allegany, February 14, 1896,—No. 906, entitled, An act to abolish the office of coroner and to provide for the performance of the duties now performed by coroners,—that deserves the careful and intelligent consideration of all citizens interested in the public welfare.

This bill was drawn, it is stated, through the collaboration of the State Bar Association, the medical societies of the State of New York, and a number of legal and medical gentlemen interested in this reform.

The object of this bill, as stated in its title, is to abolish coroners, coroners' juries, and coroners' physicians, and to create instead a medical examination system that will be removed from politics entirely, to be appointed by the several appellate divisions of the supreme court. A hearing on the bill has been set for March 4, 1896, at 2 o'clock P. M., and a full attendance of all in favor or opposed is desired. Copies of the bill may be obtained by addressing the Hon. Mr. Robbins, Assembly Chamber, Albany. We hope Buffalo will be well represented at the hearing.

---

It is now nearly two months since Professor Röntgen, of Würzburg, announced a discovery in photography that aroused the scientific world to a degree of excitement rarely before if ever equaled. It is not easy to disturb the quietude of every-day progress by even the most startling scientific discovery. We have become accustomed to such amazing advances in all directions in art and science that nothing could seemingly arrest attention, unless it were the thud of a comet striking the earth at point blank as it vaulted through space, and knocking us out of our appointed course. But the new photography of Röntgen has done it.

The newspapers and medical journals have been bristling with accounts of the application and experiments with the Röntgen rays—some in earnest and some in derision—while physicians, or more particularly surgeons, have sought to apply the principle to aid in the diagnosis of doubtful conditions. It seems likely at the present writing that a practical application of the new photography will be found in locating foreign substances, bullets, needles and such like, in the human body, but it is yet too soon to assert with positiveness just what place in medicine and surgery it will finally take. Meanwhile, the pertinent inquiry is not so much what the rays will do as, What are they ?

The *Literary Digest* has collected an excellent group of paragraphs from various sources that aim to answer this question, from which we extract the following :

Dr. Pupin, of Columbia College, in an interview with a reporter of the New York *Recorder*, said that "Dr. Röntgen's discovery, to put the whole story in a nutshell, is that the old cathode, or negative pole streamers, known to us for the last forty years, produce a strong fluorescence in a glass vacuum tube. Dr. Röntgen has discovered that, in addition to the fluorescent light, there is another form of radiation which penetrates all bodies, and casts a reflection or silhouette, varying in distinctness according to the character of the matter through which it passes, upon a sensitive photographic plate, placed beyond the object. What the possibilities of this new discovery may prove to be we cannot yet estimate. I should class it as one of the highest importance."

Prof. Edwin H. Hall, of Harvard University, in the New York *Sun*, sums up the facts concerning Röntgen's phenomena as follows :

"(*a*) That the so-called rays are sent out from the cathode of a vacuum tube, excited by a powerful alternating or rapidly interrupted current of electricity.

"(*b*) That these 'rays' act readily through wood and flesh, less readily through metals, except the lightest of metals, aluminum, and hardly at all through ordinary glass.

"(*c*) That the 'rays' are not perceptibly reflected or refracted.

"(*d*) That a medium, a solution of iodine, which absorbs the short ultra-violet rays, does not allow the Röntgen influence to pass, and a medium, a solution of alum, which absorbs long waves, does allow the Röntgen influence to pass. This is from Mr. Swinton, an English experimenter.

"(*e*) That the 'rays' are not affected by the magnet.

"The hypotheses are :

"(*a*) That the 'rays' are propagated by vibrations of greater length than those of ordinary light. Against this hypothesis we must put an experiment of Mr. Swinton.

"(*b*) That the 'rays' are ultra-violet rays. But ultra-violet rays are called such merely because they are refracted more than the violet rays, which are themselves the most refrangible rays of the visible spectrum. As the Röntgen 'rays' are apparently not refracted at all, it is difficult to see how they can be ultra-violet rays.

"(*c*) That they are rays of longitudinal vibration. It is hard to see how they would differ essentially from the electric oscillations, or displacement currents, set up in the space between the plates of an electric condenser, a Leyden jar, for instance, when the charge upon the plates is rapidly reversed. Therefore it seems that we already know something about the behavior of such vibrations, and there seems to be no reason whatever why 'rays' propagated by such vibrations should not pass with great readiness through glass. But the Röntgen 'rays' act through ordinary glass with great difficulty, and it is very doubtful whether they can be explained by means of longitudinal vibrations."

It is well to remember that the principle is yet in the infancy of its application in medicine and that published accounts of what has been accomplished through its aid should be received with caution, unless from most reliable and well-authenticated sources.

---

Dr. Henry J. Jordan, of No. 21 West Nineteenth street, New York City, who was arrested recently, charged with violating the medical law in not having registered before practising medicine, was again brought before Magistrate Cornell in the Centre street court, February 18, 1896. His bail was increased to $2,000, which he furnished, and the case will now go to the grand jury.

---

It is a pleasant indication of the appreciation of our work to find the Journal's paragraphs so frequently appropriated by other magazines; but, we submit, that it would be pleasanter if our friends would also credit us with such as are borrowed from our columns. We are pleased to observe that many of our contemporaries scrupulously obey the inexorable laws of courtesy in this respect, but, sadly enough, there are some that do not. We hope it will not become necessary to name the latter class individually.

---

The so-called Christian Scientists appear to have had a field-day with the board of aldermen at the hearing on Thursday, February 20, 1896, but if the newspaper accounts are to be relied upon, championed though they were by able counsel in the person of the Hon. D. H. McMillan, they received a pretty square knock-out from the arguments of Dr. Hopkins, who proved himself more than a match for the ex-senator on this occasion. The fact is that when a lawyer appears as the paid attorney to argue for a medical fad he is placed at a disadvantage, and so is entitled to a fair amount of sympathy, even from the side of the opposite inclining. The Journal would suggest to the senator that it would appear more in keeping with his profession, sworn officer of the courts to uphold the laws as he is, if he did not attempt to undo the effect of the recent legislation on medical questions; for this is in effect what is being attempted by him and his clients.

---

For years the medical profession of the State of New York has been struggling to obtain and maintain a medical practice law that should advance the standard of medical education. It prescribes a preliminary training for medical students, a certain course of medical study, and a final examination by the state board of medical examiners before license to practise can be obtained. Now comes Senator McMillan asking that all this be set aside in favor of a few fanatics who call themselves Christian Scientists. If Christian Scientists are to practise medicine they must do so under the statutes of the State of New York. The argument that a person has a right to worship God according to the dictates of his own conscience has nothing to do with the question. The medical profession does not challenge these well-meaning, though misguided, people on this ground, but it does insist that they shall be made amenable to the same penalties as are the doctors and other people under the ordinances for the prevention and cure of infectious diseases.

---

THIS is a very important question that cannot be brushed away with a mere breath, and we are disposed to deal with it in all seriousness. The trouble with Christian Science is, that it is neither Christian-like nor scientific. The title "C. S. D." that one of its propagandists assumes, has no meaning in law and no force in fact. It is as illegal to assume it as it would be for a man to write M.D. after his name who had never received the title from a legally incorporated medical college. It is quite time that the community aroused itself to the fact that the Health Department of a great city like Buffalo is the custodian and conservator of its sanitary affairs. It must be permitted to adopt stringent measures to prevent the spread of infectious disease, and it must prevent the sacrifice of human life when threatened through the ignorance, obstinacy, fanaticism or incompetency of its citizens. It makes no difference whether these individuals call themselves Christian Scientists or whether they assume to practise any of the other forms of quackery, and it is high time for the authorities to interfere in the name of good sense and good government.

---

THE National Public Association will meet in Buffalo next Autumn. An organization composed of scientific physicians from all parts of the continent. The purpose of the association is to devise means for the prevention of disease. Who ever heard of a national association of lawyers for the purpose of preventing litigation?

The argument that the doctors fear encroachments in their practice, hence a limitation of income, by the Christian Scientists is too silly to waste time over. In this great city, if the figures could be obtained, it would be found that the profession of medicine in its care of the poor and in gratuitous services rendered suffering humanity lends to the Lord not one-tenth but rather one-third of the fruits of the year.

The fact is our genial and talented friend, Senator McMillan, has lately ranged himself on the wrong side of all the important questions that contribute to the welfare of our city. He and his Christian Science colleagues should remember that this is near the close of the nineteenth century, and not the age of miracles. It is the iron age of positivism, and not the Utopian era.

---

The text of the ordinance proposed by the health department that has excited the ire of the Christian Scientists and their attorney is herewith submitted. We bespeak its careful reading, for we confess we are unable to discover anything in its verbiage that should not commend itself to the favor of every law-abiding citizen who is not so mentally warped by fanaticism as to be beyond the reach of reason.

Section 154. To limit the spread of contagious disease, and alleviate suffering among the poor, it is hereby provided that any minister, church member, or other person, in attendance upon the sick or injured, or who shall in any manner minister unto them, in the absence of a legally qualified practitioner of medicine, shall immediately and within six hours from first being called to attend, or from first attending any person, report such fact to the department of health, stating the name and residence of said person and the reason for such attendance.

This provision shall not apply to any case previously reported to the board of health.

A failure to comply herewith shall constitute a misdemeanor. Approved.

Charles L. Feldman, *Corporation Counsel.*
Edgar B. Jewett, }
James Mooney, } *Board of Health.*
Ernest Wende, }
*Health Commissioner.*

---

The whole issue may be stated concisely as follows : The people, through their constituted authorities, have a right to know where every case of diphtheria, small-pox or other infectious or contagious disease is located. This knowledge is necessary for the protection of the community against the spread of these diseases. This information must be lodged with the health department at the very earliest moment possible. Every citizen in such an exigency must be constituted a private detective for the time being and for the purpose named. Physicians are not exempt from this duty, but, on the contrary, are especially charged with its performance under penalty for neglect. This duty is imperative, in that public health may be maintained at the highest possible standard.

It is not class legislation to make every citizen contribute to this end; and where the laws are inadequate to accomplish it, they must be amended to meet the defect. It is specious to discuss the subject from the contrary viewpoint. This is all that is asked and the community will not be satisfied with anything else. This is the whole case in a nutshell.

---

The Stanchfield bill, which is introduced in the legislature and on which a hearing was had Wednesday, February 19, 1896, before the Public Health committee in the senate, is just now very much in evidence. Some one has asked to be defended or protected from his friends. The medical profession of the State of New York fought a hard battle to obtain a wholesome medical practice law, and now finds itself in an attitude of struggling to maintain this beneficent statute against the assault of prominent doctors who ought to be friends of the measure. At the hearing on Wednesday, however, strangely enough, out of twelve medical colleges in the state only one, Bellevue, favored the measure. Dr. E. D. Ferguson and Dr. John W. Gouley, founders and organisers of the New York State Medical Association, seemed also to be very active in the matter. Arrayed against the bill were the representatives of the medical society of the State of New York in the person of Dr. James D. Spencer, of Watertown, the president, and Dr. A. Walter Suiter, the chairman of its committee on legislation; Dr. Daniel Lewis, president of the State Board of Health; Dr. Charles E. Jones, ex-president of the State Homeopathic Medical Society; Professor Spooner, vice-president of the Eclectic Medical College; Dr. Didama, dean of the Syracuse Medical College; Dr. M. D. Mann, dean of the medical department of the University of Buffalo; Dr. W. G. Tucker, registrar of the Albany Medical College; Dr. J. H. Raymond, secretary of the faculty of the Long Island Medical College; Drs. Vander Veer and Watson, of the State Board of Regents; Dr. Eugene Beach, of the State Board of Medical Examiners; Professors Ward and Macdonald, of the Albany Medical College, and many others of prominence in the profession, representing all the schools of practice.

Each of the representatives of the various colleges present in turn pleaded for the existing law and spoke in terms of highest commendation of the excellent management of the Regents' office. The College of Physicians and Surgeons, of New York City—the medical department of Columbia University—sent in a protest against the bill signed by every member of its faculty. Physicians from all parts of the state sent words of protest against any alteration of the law, such as is contemplated in the Stanchfield bill. Prominent educators, including men like President Low, of Columbia, wrote the chairman of the committee, protesting against the proposed changes. We do not believe this dangerous bill will be allowed to pass the legislature, but should it so happen we are firmly of the opinion that Governor Morton will make short work in vetoing it when it comes before him.

---

A HEARING was held at the Fifth Avenue Hotel in New York, Saturday, February 15, 1896, before a committee of the State Medical Examining and Licensing Board, to determine the propriety of dividing the examinations for license. The friends of the proposed plan claim that students should be examined in the primary subjects, such as anatomy, physiology and chemistry as soon as they receive their pass-cards on these subjects in the colleges, and thus be left free to pursue the secondary, or practical branches during the remainder of their student life. The opponents of the plan consider the State examination as one simply to determine the equipment of the candidate for the practice of medicine, and not to be of a technical nature.

The committee, consisting of Dr. George R. Fowler, of Brooklyn, chairman; Dr. William Warren Potter, of Buffalo; and Dr. M. J. Lewi, of New York, secretary, listened to arguments pro and con for nearly three hours, and will make up their report on the subject as soon as possible. This will be transmitted finally to the regents for such action as they may deem proper.

---

THE New York State Civil Service Commission finds great difficulty in securing suitable candidates for the position of woman physician in the State hospitals. These positions are desirable ones, paying from $1,000 to $1,500 per year, besides giving ample opportunity for practice and study in nervous and mental diseases, with pleasant home and associations. The examination advertised in January failed for lack of applicants.

This appears to be one of the places that women are not seeking, but in view of its advantages, it is difficult to understand the reason why it is allowed to go begging.

---

The condition of the water-supply of Buffalo, or rather the lack of supply, is a disgrace to the civilisation of the period. With an abundance of pure water within easy reach, with a knowledge of the engineering methods that should bring it permanently into every consumer's faucets, with an abundance of money to accomplish the feat, why on earth the inhabitants of this great city should be treated to a water famine by the board of public works, is a conundrum that we shall be glad to have somebody satisfactorily answer! One thing is certain: the people will not tolerate a repetition of the incompetence that has been manifested in the past. Pure water and plenty of it is a basic element in the sanitary economics of populous cities, and the people will have it or invoke retributive justice on the heads of those who are directly responsible for a failure to supply it. And they will not have it measured out to them through water-meters either!

---

